# Mitochondrial content is central to nuclear gene expression: Profound implications for human health

**DOI:** 10.1002/bies.201500105

**Published:** 2016-01-03

**Authors:** Rebecca Muir, Alan Diot, Joanna Poulton

**Affiliations:** ^1^ Nuffield Department of Obstetrics and GynaecologyUniversity of OxfordJohn Radcliffe HospitalOxfordUK

**Keywords:** ageing, gene expression, gene regulation, mitochondria, mitochondrial biology, mtDNA, single cell analysis

## Abstract

We review a recent paper in Genome Research by Guantes et al. showing that nuclear gene expression is influenced by the bioenergetic status of the mitochondria. The amount of energy that mitochondria make available for gene expression varies considerably. It depends on: the energetic demands of the tissue; the mitochondrial DNA (mtDNA) mutant load; the number of mitochondria; stressors present in the cell. Hence, when failing mitochondria place the cell in energy crisis there are major effects on gene expression affecting the risk of degenerative diseases, cancer and ageing. In 2015 the UK parliament approved a change in the regulation of IVF techniques, allowing “Mitochondrial replacement therapy” to become a reproductive choice for women at risk of transmitting mitochondrial disease to their children. This is the first time that this technique will be available. Therefore understanding the interaction between mitochondria and the nucleus has never been more important.

**Abbreviations**
AS: alternative splicing
GWA: genome wide association
GWAS: genome wide association studies
HIF: hypoxia inducible factor
OXPHOS: oxidative phosphorylation
SDH: succinate dehydrogenase
SNPs: single nucleotide polymorphisms
TCA cycle: the citric acid cycle

## Introduction

Guantes et al. 1 begin by observing that gene expression is noisy in a population of genetically identical cells, and that this has consequences for cell behavior 2. Something else must be affecting expression, either downstream or upstream. Reasoning that gene expression uses a high proportion of cellular energy (indeed, 75% in *E. coli* 3), the authors demonstrate that the cellular content of mitochondrial proteins seems to exert far‐reaching effects on nuclear gene expression. They previously showed that heterogeneity in the volume of mitochondria in individual cells is a source of variability in transcription rate 2, which in turn is highly sensitive to the concentration of ATP. This links transcription to mitochondrial function 2. In the current study, Guantes et al. attribute the cause of variability in global gene expression to energetic modulation by the mitochondrial network 1. They start by showing that mitochondrial mass, measured as the integrated signal of a mitochondrial dye called CMXRos, is proportional to the abundance of a number of mitochondrial proteins involved in energy production, referring to this as “mitochondrial content”. They measure the mitochondrial content of individual cells together with either (a) the protein content, (b) the RNA polymerase II content/activity, or (c) the chromatin modification. They then investigate the variation independent of the mitochondria, and the co‐variation with mitochondria; finally they calculate the mitochondrial contribution to the variability of (a), (b), and (c).

**Figure 1:**
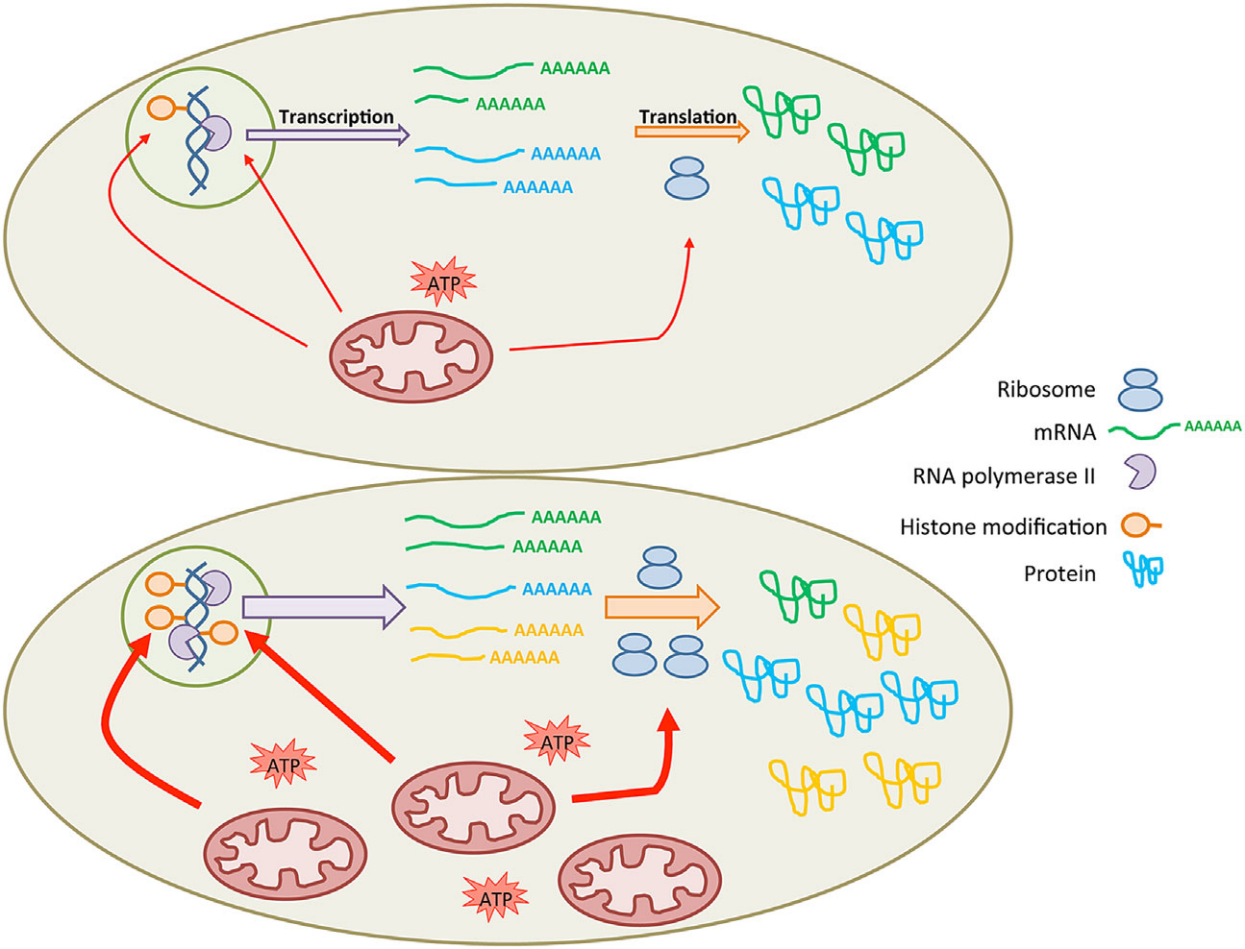
Guantes et al. have shown that mitochondria are an important factor influencing the cell's protein content variability. Mitochondria affect the histone profile, the transcription level, the alternative splicing, and the mRNA translation into proteins (upper panel). An increase in mitochondrial content, i.e. ATP content, of the cell will lead to an activation of the chromatin by histones acetylation and methylation, a greater number of genes transcribed by the RNA Polymerase 2, a different pattern of alternative splicing and a higher number of ribosomes for protein translation (lower panel). This results in different sets of proteins between cells with a high content of mitochondria and cells with a lower content of mitochondria.

Their results show that mitochondrial content accounts for half of the protein variability in a cell population. This genome‐wide effect is due to an effect at four different levels of the gene expression: 1) on chromatin activation via the acetylation of histones; 2) on transcription activity, 3) on alternative splicing (AS), 4) on protein synthesis. This is illustrated in Fig. 1.

All of these effects are likely to be due to the ATP levels linked to the mitochondrial content and function of the cell. Although it seems counterintuitive to think that a global constraint such as energy can be responsible for the substantial individual variation between cells, it is apparent that mitochondria are heterogeneous in structure, number, and function across a population of cells, especially in pathological states. It follows that energy production will be heterogeneous. Gene expression has multiple steps with very different energy demands along the way. Mitochondrial variation is likely to play a central role in diseases because of its striking effects on the transcriptional profile of cells when different levels of mutant mtDNA are present 4.

ATP levels are often used to assess mitochondrial function, but this is problematic because homeostatic mechanisms obscure changes. ATP flux is a better reflection of changes in mitochondrial function, but it is difficult to measure accurately. This limitation has masked the central role of mitochondrial variation. Most of the data implicating mitochondria in disease arise from genetic studies of mitochondrial DNA (mtDNA). Hence, the methodology of Guantes et al. is of particular note because of the primary focus on mitochondrial mass and gene expression, rather than on ATP/mtDNA.

This paper is also one of the first to describe non‐genetic variability in human cell lines that exhibit characteristics of human cancer and disease. We hypothesize that the differences in gene expression have important implications for multifactorial disease and cancer. Additionally, we believe that understanding the mitochondrial influence on gene expression adds another dimension to the social and ethical issues associated with recent advances in nuclear transfer techniques.

## Mitochondria are responsible for nuclear genome complexity

The nuclear and mitochondrial genomes in eukaryotes have always been co‐dependent, essentially guiding each other's evolution through deep time as a result of the original endosymbiosis 5. In the eukaryotic cell, mitochondria produce energy via oxidative phosphorylation (OXPHOS), a chain of chemical reactions resulting in ATP production. Unlike chromosomal genes, mitochondrial genes are packed into a tiny circle of DNA containing 13 protein reading frames encoding OXPHOS‐related proteins. OXPHOS is hypothesized to be integral to the evolution of multicellular life, because cells with a tiny energy budget have to restrict their genome size and complexity 3. From the point of view of a cell, the expression of genes is enormously expensive, and yet it is both greater and more diverse in eukaryotes compared to prokaryotes.

The reciprocal interaction between mitochondria and the nuclear genome is archaic and highly evolved 3, leading Guantes et al. to explore the mitochondrial influence on nuclear gene expression, characterizing the impact that mitochondria have on nuclear genes from a quantitative perspective.

Differences in genetically identical cell populations 6 are found even when grown in identical environments 7. Previous research has shown that gene and protein expression levels can vary significantly among the same cell population with different functional results 8, 9, but Guantes et al. have gone a step further by using single‐cell analysis with a focus on gene expression and mitochondrial content. Single‐cell analysis is now a crucial tool for understanding the complexity behind variations in cell populations. Genetic and phenotypic heterogeneity among cells is the rule, not the exception 10. Much of the literature has focused on microbes rather than human cells. Therefore this paper is an important step towards identifying the causes of noise in human disease 11.

Mitochondria have been implicated in cancer and a wide range of metabolic and degenerative diseases, including ageing itself. In many cases the links are apparent but incomplete. The new findings presented by Guantes et al. have the potential to fill gaps across the board because of their focus on several levels of gene regulation 1. First we will highlight how these regulatory mechanisms are influenced by the mitochondria. Then we will discuss how mitochondria are implicated in cancer, multifactorial disease, and neurodegenerative disease, offering our interpretation of how the research of Guantes et al. can be applied to these situations.

## Mitochondria drive alternative splicing

AS is crucial to Iborra and co‐workers main finding that mitochondria contribute over half of protein diversity in the cell. AS allows the economical storage of genetic information, and can result in more transcripts than the number of genes in a human's entire collective genome 12. Although this mechanism allows tissue specificity and evolutionary flexibility, the high potential for protein diversity brings with it the potential for malignancy. A common theme in the “hallmarks of cancer” is a switch in splicing patterns associated with a more aggressive, invasive cancer phenotype. Misregulation of an angiogenesis splice form has been found, as have splice forms that contribute to resistance to apoptosis 13. Diseases associated with AS are well‐documented 14, 15. Guantes et al. postulate that mitochondria will affect AS either through impacting the energy supply of the AS molecular machinery, or indirectly through mechanisms that are highly energy dependent and play a role in AS, such as the epigenetic mechanisms of chromatin remodeling 16.

## Mitochondria influence epigenetic chromatin modifications

Epigenetic modification is one of the regulatory steps in gene expression, and its mechanisms fall broadly into three categories: methylation, histone modification, and RNA‐associated silencing 17. Chinnery et al. speculated that the varying severity seen in patients with mitochondrial disease might be partly explained by the downstream effects caused by modification of nuclear DNA 18. Guantes et al. provide evidence that mitochondria play a role in epigenetic modification: they discovered three histone modifications that co‐vary with mitochondrial content and are linked with chromatin activation, namely H4K16 19, H3K4me3, and H3K36me2 20. However, they did not find links with histone modifications involved in chromatin repression. Such acquired epigenetic changes 21 may link environmental risk factors to important diseases including human cancer. Guantes et al. also comment that the mitochondria provide metabolites needed for epigenetic modification, and therefore that a lack of mitochondrial metabolites may be rate‐limiting and affect the frequency of nuclear gene modification 22. Their findings add substance to the notion of a “mitocheckpoint” in which damage to mitochondria affects the availability of S‐adenosyl methionine, thus modulating methylation of the nuclear genome 23. Although this field is in its infancy, mitochondrial epigenetics may contribute to the effect that variance in mitochondrial number and functionality has on the nucleus, affecting penetrance of disease 13.

## Mitochondria regulate expression of cancer genes

After Otto Warburg observed that cancer cells rely on glycolysis instead of OXPHOS for energy production 24, mitochondria have been thought to be implicated in carcinogenesis. Later discoveries continued to place mitochondria at the forefront of cancer research as it was found that nuclear‐mitochondrial and mitochondria‐to‐nucleus signal integration, control of apoptosis, and various metabolic pathways were under mitochondrial control. Notably, up‐regulation of hypoxia inducible factor (HIF) and of HIF‐responsive genes is a feature of many cancers. Rarely, mutations in genes encoding TCA cycle enzymes 25, such as the mitochondrial complex succinate dehydrogenase (SDH) 26, are central to neoplastic transformation. MtDNA analysis reveals that most or all cancers accumulate somatic mitochondrial as well as nuclear genome mutations 27. The origins and impact of cancer‐associated mutations in mtDNA were unclear, and some investigators therefore suggested that abnormal mitochondrial function might cause oncogenic transformation. If this were widely applicable, therapeutic approaches should aim to augment and not inhibit mitochondrial function 28.

However, recent sequencing that extensively explores the somatic alterations in the mitochondrial genomes of cancers suggests that the reverse is true for the majority of cancers. Ju et al. found that most mitochondrial genome mutations have no discernible effect 29. The apparent high frequency of “passenger” mtDNA mutations in tumors could arise from their stochastic accumulation. Furthermore, DNA mutations that damage normal mitochondrial activity were less likely to be maintained in cancer cells. While there are undoubtedly additional metabolic alterations and dependencies of cancer cells that may be exploited to improve anticancer therapy, it is clear that most cancers need their mitochondria for proliferation, maintenance and invasive behavior. The increased risk conferred by impaired SDH activity is therefore the exception and not the rule 26. Hence for most tumor types, these new findings suggest that drugs that impair mitochondrial function may slow down cancer progression by influencing nuclear gene transcription. Studying the mitochondrial activity and transcriptional profile of primary and cancer cell lines will help clarify the relationship between mitochondria and cancer cell behavior. Further work is needed to determine how this relates to cancers that arise from dysregulated stem cells.

## Mitochondria regulate genes in multifactorial disease

Chromosomes segregate in accordance with Mendel's laws, and in this process, genes are subject to rigorous selection pressures, leading to disease‐causing genes being eliminated from the population. Mitochondrial genes work differently. There are thousands of copies of mtDNA in every nucleated cell, and these have a high mutation rate, and a maternal mode of inheritance. Furthermore, healthy and mutant mtDNA are able to co‐exist in the same cell, in a state called heteroplasmy. The “Mendelian disease” paradigm led to Genome‐Wide Association Studies (GWAS) gaining immense popularity within genetics research. While this generated useful results for some diseases, many other diseases remain relatively intractable 30. This may be because the mechanism behind many diseases is actually bioenergetic, given the strong links between mtDNA and some types of disease 31. The supposedly “genome‐wide” GWAS techniques typically did not even consider mitochondrial DNA. To overcome this failing, GWAS investigators then set out to explore the effects of mtDNA single nucleotide polymorphisms (SNPs) in multifactorial diseases. However, several seemingly well‐designed studies failed to detect associations between disease‐associated variants located in the mitochondrial genome and diabetes 32, 33, 34.

This is because mtDNA poses several problems. Firstly, these studies could have missed heteroplasmic mtDNA mutants in blood, which often do not reflect the load in tissues, such as the insulin‐secreting β cells of the pancreas. In β cells they may reach levels sufficient to change transcriptional profile while blood levels may decline to undetectable levels 35. Secondly, a reproducible association may be overlooked if SNPs are considered as independent entities, the default position for GWAS studies. There are several examples where the context of mtDNA variants may affect the risk of disease that they confer 36, 37. Independence of SNPs is less appropriate for mitochondrial than for nuclear SNPs, because intermolecular recombination is negligible in the context of maternal inheritance. In some 36, 37 but not all 38 such examples, the group of specific mtDNA SNPs implicated in conferring risk of disease have been identified. One way to probe groups of SNPs, which co‐segregate because they are ancient associations, is to consider mtDNA haplogroups (in which people of different geographical regions have mtDNA variants which are population‐specific) 39. However, mtDNA haplogroups may be less helpful 40, 41 for rapidly mutating susceptibility variants 42 such as the OriB variant 43. This variant is situated within an origin of replication (OriB) 44. It lies within a homopolymeric C tract in the D‐loop region of the mitochondrial genome. It is unique because SNPs in the local mtDNA sequence that affect the risk conferred interrupt the homopolymeric tract and compromise its secondary structure. Intuitively such changes should affect the function of the origin of replication, potentially being mildly deleterious 45. We demonstrated a significant association between sequence variation around OriB and type 2 diabetes, initially in a UK population 40. Despite being missed in the large GWAS studies 32 the association was confirmed in a large meta‐analysis of Europid 43 and other populations 45 that took the local sequence into account. This variant predisposes to thinness, both at birth 46 and in young adults 47, helping to explain the link between low birth weight and diabetes.

Hudson et al considered haplotypes when they re‐investigated mtDNA SNPs in 38,638 individuals with 11 major late‐onset diseases and 17,483 controls in a GWAS study 39. Their data suggests that deleterious variants that are associated with one or more common diseases are escaping selection. Like the OriB variant (which was not investigated in their study), many of these mutations emerged recently. They postulated that these recent mutations interact with nuclear loci and modify the risk of developing multiple common diseases. In line with Iborra's findings, these apparently detrimental variants could impair global nuclear gene expression, potentially contributing significantly to the pathogenesis. The next step will be to investigate single cell transcripts and protein profiles relative to heteroplasmic variants, and the protein profile in the OriB variant compared to controls.

## Mitochondria regulate genes associated with degenerative diseases and ageing

Mitochondria are thought to play an important role in neurodegeneration and ageing. Significantly, Attardi showed that fibroblast respiratory chain function declines with age 48. Several authors reported accumulation of low levels of mtDNA rearrangements (the “common deletion”) in Alzheimers 49, Parkinson's disease 50 and ageing 51, in failing and ageing hearts 52, 53, and even in human oocytes 54. This led to the “vicious circle” theory of mitochondrial ageing, in which somatic mutation of mtDNA engenders respiratory chain dysfunction, enhancing the production of DNA‐damaging oxygen radicals and hence mtDNA damage 55. However, the levels of mutant mtDNA detected were often extremely low (<1%) compared with the levels found in bonafide mitochondrial disease 56. Such levels are more likely to reflect an underlying problem than to be damaging in themselves. More recent work on neurodegeneration implies disturbances in cellular and organellar quality control mechanisms and in mitochondrial dynamics (mitochondrial shape, size, distribution, movement, and anchorage). Mitochondrial dynamics are central to mitophagy (the type of quality control among several that is most likely to maintain mtDNA), and genes required for mitophagy are involved in familial Parkinson's disease 57. We recently showed that fibroblast mitophagy declines with the age of the donor 58. This again implicates declining mitochondrial function as important in the ageing process, and potentially explains the accumulation of mtDNA deletions in human ageing. There are additional reasons for thinking that energy metabolism might be important in neurodegeneration: for instance, more gene products implicated in neurodegeneration are targeted to the mitochondria than expected by mere chance alone (23% compared with 8%) 59. Hence mitochondrial defects may be important in neurodegenerative disorders.

The most convincing recent evidence that mitochondria are involved in ageing came from studies of mtDNA maintenance. Larsson and co‐workers generated a mouse with a proofreading defect in polymerase gamma, a protein that is essential for all mitochondrial DNA replication. Unexpectedly the main phenotype of this mouse was premature ageing 60, and this positioned the accuracy of mitochondrial DNA replication at the center of the ageing debate. Subsequent studies, however, did not support a simplistic interpretation of the data largely because of the inadequacies of the hypotheses being tested 61. Even if mitochondrial damage were important, how did this translate into effects on ageing? Reactive oxygen species, an essential component to the vicious circle of ageing did not accumulate 62. Neither did the common deletion, widely considered to be a hallmark of mitochondrial DNA damage in humans 63. Iborra's hypothesis that mitochondria are central to gene expression provides a first step. But if a decline in oxidative phosphorylation were sufficient to cause premature ageing, should it not be a common feature of mitochondrial disease? Rather, the between‐cell variability in mitochondrial function present in this mouse, which is already known to have profound effects 64, is the more plausible answer. Again, use of the impressive range of single cell techniques that are now available to explore these questions will ultimately confirm or refute this claim. Indeed, investigating the activity level of telomerase in cells with a high content of mitochondria or ATP and conversely in cells with less energy could bring together the role of mitochondria and telomerase in ageing. Given our results showing a decrease in mitochondrial quality control with age, the older cells may have an altered energetic status and thus differ in net protein activities, among them telomerase.

## Social and ethical insights

In this essay we have seen that mitochondria alter the nuclear gene expression profile of cells. This concept takes its lead from the knowledge that a high amount of mtDNA sequence variance exists in the human population 65 and that over evolutionary time, mitochondrial‐nuclear interactions become highly specific 66. These findings are important for understanding the ethical ramifications of the UK Parliament approval of “mitochondrial donation” or “mitochondrial replacement therapy” 67, 68, 69. These terms describe an in vitro fertilization‐based technique for reducing the dose of mutant mtDNA in the pre‐implantation embryo. Successful experiments have been carried out in mice 70 and monkeys 71 and are technically possible in human embryos 72. Concerns have been expressed that nuclear gene expression may need to be compatible with the mtDNA of the donor 73, and that the mtDNA of the donor should be closely related to that of the recipient 68, 74. However, the concern raised by the findings of Guantes et al., that the mitochondria themselves may be important regulators of nuclear genes, has barely been considered. The view that “all you need is enough energy” and that mitochondrial transfer is equivalent to “changing a laptop battery” is inadequate 67. Human development involves a carefully choreographed sequence of expressed genes, and in the case of the respiratory chain, these are encoded by both nuclear and mtDNA. As Guantes et al show, ATP is a key regulator of gene expression. Fundamentally altering the energetics is likely to affect the balance of the complex processes underlying health and personality. The UK parliament was briefed with the information that “Mitochondria have their own separate DNA, which carries just a few genes. All of these genes are involved in energy production but determine no other characteristics. And so, any faults in these genes lead only to problems in energy production… all available scientific evidence indicates that the genes contributing to personal characteristics and traits come solely from the nuclear DNA” (http://www.hfea.gov.uk/docs/2014-10-01_Mitochondrial_donation__an_introductory_briefing_note_-_final.pdf). However, the data presented by Guantes et al suggest that mitochondria are determinants of important traits including multifactorial disease, cancer, and ageing. Mitochondrial replacement therapy may well be a viable strategy for fixing the energy deficits in mitochondrial disease, but the donated mitochondria are likely to influence other characteristics. In future, regulators would be well advised to ensure that the mitochondrial and nuclear genomes of donor and recipient are sufficiently matched, as this may be critical to the success of mitochondrial replacement therapy 68, 73, 74.

## Conclusions and outlook

Guantes et al. have begun to explain the genetic mechanisms by which ATP alter the cell, namely epigenetics and alternative splicing, and we have shown that their central tenet can be applied to established theories in cancer metabolism and ageing. This research has revealed that the mitochondria, the ancient organelles once seen simply as generators of ATP, affect cellular longevity and health by affecting nuclear transcription and translation. Future work should focus on verifying the links between the energetic status of the cell and its individual portfolio of expressed genes in health and disease. Outcomes of such studies might open promising new therapeutic strategies. Moreover, they will improve the safety of mitochondrial replacement therapy by helping to clarify the cross‐talk between mitochondria and the nucleus.

The authors have declared no conflict of interest.
